# Detection of Histone H3 mutations in cerebrospinal fluid-derived tumor DNA from children with diffuse midline glioma

**DOI:** 10.1186/s40478-017-0436-6

**Published:** 2017-04-17

**Authors:** Tina Y. Huang, Andrea Piunti, Rishi R. Lulla, Jin Qi, Craig M. Horbinski, Tadanori Tomita, C. David James, Ali Shilatifard, Amanda M. Saratsis

**Affiliations:** 1 Division of Pediatric Neurosurgery, Department of Surgery, Ann & Robert H. Lurie Children’s Hospital of Chicago, Chicago, IL USA; 2 Department of Neurological Surgery, Northwestern University Feinberg School of Medicine, Chicago, IL USA; 3 Department of Biochemistry and Molecular Genetics, Northwestern University Feinberg School of Medicine, Chicago, IL USA; 4 Division of Hematology, Oncology, Neuro-Oncology & Stem Cell Transplantation, Ann & Robert H. Lurie Children’s Hospital of Chicago, Chicago, IL USA; 5 Department of Pathology, Northwestern University Feinberg School of Medicine, Chicago, IL USA

**Keywords:** Cerebrospinal fluid, Liquid biopsy, Diffuse midline glioma, Diffuse intrinsic pontine glioma (DIPG), H3K27M

## Abstract

Diffuse midline gliomas (including diffuse intrinsic pontine glioma, DIPG) are highly morbid glial neoplasms of the thalamus or brainstem that typically arise in young children and are not surgically resectable. These tumors are characterized by a high rate of histone H3 mutation, resulting in replacement of lysine 27 with methionine (K27M) in genes encoding H3 variants H3.3 (*H3F3A*) and H3.1 (*HIST1H3B*). Detection of these gain-of-function mutations has clinical utility, as they are associated with distinct tumor biology and clinical outcomes. Given the paucity of tumor tissue available for molecular analysis and relative morbidity of midline tumor biopsy, CSF-derived tumor DNA from patients with diffuse midline glioma may serve as a viable alternative for clinical detection of histone H3 mutation. We demonstrate the feasibility of two strategies to detect H3 mutations in CSF-derived tumor DNA from children with brain tumors (*n* = 11) via either targeted Sanger sequencing of *H3F3A* and *HIST1H3B,* or *H3F3A* c.83 A > T detection via nested PCR with mutation-specific primers. Of the six CSF specimens from children with diffuse midline glioma in our cohort, tumor DNA sufficient in quantity and quality for analysis was isolated from five (83%), with H3.3K27M detected in four (66.7%). In addition, H3.3G34V was identified in tumor DNA from a patient with supratentorial glioblastoma. Test sensitivity (87.5%) and specificity (100%) was validated via immunohistochemical staining and Sanger sequencing in available matched tumor tissue specimens (*n* = 8). Our results indicate that histone H3 gene mutation is detectable in CSF-derived tumor DNA from children with brain tumors, including diffuse midline glioma, and suggest the feasibility of “liquid biopsy” in lieu of, or to complement, tissue diagnosis, which may prove valuable for stratification to targeted therapies and monitoring treatment response.

**Electronic supplementary material:**

The online version of this article (doi:10.1186/s40478-017-0436-6) contains supplementary material, which is available to authorized users.

## Introduction

Diffuse midline gliomas are high-grade glial neoplasms of the thalamus or brainstem (including diffuse intrinsic pontine glioma, DIPG), and occur almost exclusively in young children. These tumors are not surgically resectable due to their anatomic location, which limits tissue available for diagnosis and molecular study. However, recent studies have revealed molecular characteristics of diffuse midline gliomas that are distinct from hemispheric pediatric and adult gliomas. Specifically, recurrent somatic mutations in genes encoding the replication-independent histone H3 isoform, H3.3 (*H3F3A*), and the replication–dependent isoform, H3.1 (*HIST1H3B*), are reported in a majority of pediatric midline and high-grade gliomas [10, 20, 33, 38]. These mutations result in lysine 27 to methionine substitution (H3.3 or H3.1 K27M) or glycine 34 to valine or arginine substitution (H3.3 G34V/R) The K27M mutation is observed in up to 80% of diffuse midline gliomas, and G34V/R mutations occurs in up to 30% of hemispheric pediatric gliomas [10, 20, 33, 38]. Because K27 and G34 are located in the N-terminal tail of the Histone H3 protein, these amino acid residues are critical sites for post-translational histone modification [28]. As a result, H3K27M and H3G34V mutations have a significant impact on regulation of gene transcription and DNA methylation [2, 27, 31, 32]. Because patients with H3 mutations demonstrate a more aggressive clinical course and poorer overall response to therapy [3, 16, 20], the biological effects H3 mutations are thought to contribute to the lack of clinical response to treatments that are more effective in H3 wild type gliomas [16].

Given the biological and clinical implications of histone H3 mutation in diffuse midline glioma, mutation detection is of great interest for advancing understanding of tumor biology and improving patient treatment. However, biopsy of these tumors for genetic analysis is not without clinical risk [30]. In contrast, cerebrospinal fluid (CSF) is more easily obtained than midline brain tumor tissue, and tumor-specific genetic alterations may be detected in CSF due to direct contact with brain tumor tissue [5, 14, 26, 37]. CSF from midline glioma patients may therefore serve as a reasonable alternative for detection of these mutations without the risk of tissue biopsy. Therefore, we set to detect Histone H3 mutation in archival CSF collected from pediatric patients with diffuse midline glioma, including DIPG, and to validate these findings in patient-derived tumor tissue. This approach could serve as a safe and robust method of “liquid biopsy” for histone H3 mutation detection in children with diffuse midline glioma, to potentially facilitate clinical stratification to targeted therapies and measure response to treatment.

## Materials and methods

### CSF and tissue specimen collection

CSF specimens were collected from children with brain tumors during the course of treatment (*n* = 11), either upon placement of a CSF diversion device (ventricular shunt, external ventricular drain (EVD) or indwelling CSF reservoir, 2/11, 18%), or via sterile access of an existing CSF diversion device (ventricular shunt or CSF reservoir, 9/11, 82%). CSF collected via ventricular shunt tap from a child with congenital hydrocephalus was also used as a negative control. When available, fresh frozen (*n* = 2) and paraffin embedded tumor tissue (*n* = 6) were used to validate CSF sequencing results. Tumor tissue specimens (*n* = 8) were acquired either during the course of treatment at the time of tumor resection or biopsy (7/8, 87.5%) or postmortem (1/8, 12.5%). Informed consent for specimen analysis was obtained under protocols approved by Ann & Robert H. Lurie Children’s Hospital of Chicago and Northwestern University Institutional Review Boards (Lurie 2012–14877 and 2005–12252, NU STU00202063). All patient identifiers were removed at the time of specimen collection and a numerical identifier was assigned to each specimen before processing (Table 1).

**Table 1:** Biological Specimens Evaluated for H3 Mutation

	Patient Information		CSF Collection		Carrier		CSF DNA yield (ngDNA/μLCSF)	CSF DNA Analysis			Tissue DNA Analysis		CSF & Tissue analyses concordance on H3.3K27M status
	ID	Histologic Diagnosis	Source	Time of Acquisition	yRNA	LPA		H3.3 Sanger Seq	H3.3 Mutation-Specific PCR	H3.1 Sanger Seq	IHC	H3.3 Sanger Seq	
Expected to harbor K27M	1	DIPG	R	At Reservoir Placement	–	Y	0.01	–	K27M*	–	Tissue Not Available		–
	2	DIPG	R	Reservoir Tap During Treatment	Y	Y	0.05	K27M*	K27M^	–	Tissue Not Available	K27M^	Y
	3	DIPG	R	Reservoir Tap During Treatment	–	Y	0.28	WT	WT	WT	Tissue Not Available		–
	4	DIPG	EVD	At Tumor Biopsy	–	Y	0.09	K27M*	K27M^	–	K27M*	K27M^	Y
	5	Thalamic Anaplastic Astrocytoma	EVD	At Tumor Biopsy	–	Y	0.03	–	K27M*	–	K27M^	–	Y
	6	Thalamic Anaplastic Astrocytoma	R	At Reservoir Placement	–	Y	0.02	Insufificient DNA	Insufificient DNA	Insufificient DNA	WT	–	–
Not Expected to harbor K27M	7	Thalamic Pilocytic Astrocytoma	R	At Reservoir Placement	–	Y	0.14	WT	–	Insufificient DNA	Tissue Not Available		–
	8	Supratentorial Glioblastoma	R	At Reservoir Placement	Y	Y	1.28	G34V	–	–	WT	–	Y
	9	Cerebellar Juvenille Pilocytic Astrocytoma	EVD	At Tumor Resection	–	Y	0.40	WT	–	Insufificient DNA	WT	–	Y
	10	Right Lateral Ventricular Choroid Plexus Papilloma	EVD	At Tumor Resection	Y	Y	3.76	WT	–	WT	WT	WT	Y
	11	Medulloblastoma with 4th Ventricular Extension	EVD	At Tumor Resection	Y	Y	0.80	WT	–	Insufificient DNA	WT	WT	Y
Control	12	Congenital hydrocephalus	S	Shunt Tap During Treatment	–	Y	0.04	–	WT	–	–	–	–

Cerebrospinal fluid (CSF) collected from children with brain tumors (*n* = 11), and shunted congenital hydrocephalus with no brain tumor history (*n* = 1), was evaluated for H3 mutation via *H3F3A* (H3.3) and *HIST1H3B* (H3.1) sequencing. All CSF specimens were derived from the lateral ventricle via an implanted CSF reservoir (R), shunt (S), or external ventricular drain (EVD). Available matched tumor tissue (*n* = 8) was analyzed via Sanger sequencing and/or tissue immunohistochemical staining in order to validate CSF sequencing results. KEY: * = first detection of K27M mutation; ^ = validation of K27M mutation; WT = wild type

### Extraction of DNA from CSF

CSF specimens were immediately placed on wet ice upon collection then stored at −80 °C. In order to eliminate genomic DNA (gDNA) derived from white blood cells, which can potentially interfere or mask the signal of tumor-derived DNA [26], CSF specimens were centrifuged at 500 × *g* for five minutes at 4 °C within two hours of collection to isolate the cell-free supernatant, which has been shown to yield DNA for subsequent extraction and sequencing [18]. DNA was extracted from 0.4 to 2 mL CSF (mean = 1.1 mL, standard deviation = 0.65 mL) using the QIAmp Circulating Nucleic Acid Kit (Qiagen). Extraction was performed per manufacturer’s protocol with the provided carrier RNA, as well as with 15 μg/mL linear polyacrylamide (Ambion/Applied Biosystems) to precipitate DNA fragments >20 base pairs [4, 21]. Briefly, 100 μL proteinase K, 0.9 mL lysis buffer ACL, and either 1 μg carrier RNA or 15 μg LPA was added to every 1 mL CSF. After a 30-min incubation at 60 °C, 1.8 mL binding buffer ACB was then added, and the mixture was incubated on ice for five minutes. The lysate-buffer mixture was passed through the supplied minicolumn, washed with washing buffers and DNA eluted with 30 μL buffer AVE.

### Evaluating size distribution of extracted DNA fragments

To examine the effect of CSF centrifugation on fragment distribution of extracted DNA, equal volumes of CSF specimens were spun under the following three conditions: no spin, spin at 500 *g* × 5 min, and spin at 1000 *g* × 10 min based on a published protocol for ctDNA isolation from CSF [26]. DNA were then isolated from these CSF specimens using the method above. Fragment size distribution of extracted DNA was evaluated by loading 1 μL of DNA onto the Agilent 2100 Bioanalyzer.

### Extraction of DNA from brain tumor tissue

For DNA extraction from fresh-frozen paraffin embedded (FFPE) tissue, four 20 μm sections were de-paraffinized via four rounds of xylene incubation, followed by rehydration with serial ethanol incubation at decreasing concentrations [7] (100%, 95%, 70%, 50%, 20% ethanol, and water). Extracted chromatins were then sonicated using E220 focused-ultrasonicator (Covaris) for 30 min at 20% duty cycle, 175 peak intensity power, 200 cycles per burst. Sonicated DNA fragments were then purified with the QIAmp Circulating Nucleic Acid Kit (Qiagen) using the method described above. This FFPE tissue sonication protocol was chosen to remain consistent with FFPE ChIP-Seq protocols used by our group in order to ensure the ability to perform ChIP-Seq on these specimens in future studies. Fresh frozen tumor tissue was used for DNA extraction using the DNeasy Blood & Tissue Kit (Qiagen) per manufacturer’s protocol. Briefly, 180 μL buffer ATL and 20 μL proteinase K were added to the tissue specimen. After a ten minute incubation at 56 °C, 200 μL buffer AL and 200 μL ethanol were added. The mixture was then passed through the supplied spin column and washed with buffers AW1, AW2, and finally eluted with 100 μL of buffer AE. Extracted DNA was stored at −20 °C.

### Extraction of DNA from pediatric glioma cell lines

Genomic DNA was isolated from H3.3K27M mutant DIPG and H3K27 wild type pediatric glioma primary tumor cell lines (SF8628 and SF9427, respectively). Cell lines were established as previously described [22] and obtained from the University of California, San Francisco (UCSF) medical center in accordance with institutionally approved protocols. SF8628 was cultured in media containing 1× DMEM (Thermofisher), 10% FBS (Corning), 1× glutamine (Life Technologies), 1× penicillin/streptomycin (Life Technologies). SF9427 was cultured in media containing 1× Neurobasal-A, 1× DMEM/F12, 10 mM HEPES, 1 mM MEM Sodium Pyruvate, 1× MEM Non-Essential Amino Acids, 1× glutamine, and 1× penicillin/streptomycin (all Invitrogen). Both cell lines were grown in 5% CO_2_ at 37 °C. The QIAamp DNA Mini Kit (Qiagen) was used to extract genomic DNA from 4.4x10^6^ cells per manufacturer’s protocol. Briefly, cells were harvested using a cell scraper and centrifuged for five minutes at 300 × *g*. Supernatant was removed and the cell pellet resuspended in 200 μL PBS. 200 μL buffer AL and 20 μL proteinase K was added and the mixture incubated for ten minutes at 56 °C. 200 μL ethanol was added and the mixture passed through the supplied spin column, followed by two rounds of washing with AW1 and AW2. Finally, 150 μL buffer AE was used to elute the DNA.

### Targeted Sequencing of *H3F3A* and *HIST1H3B*

**Fig. 1:**
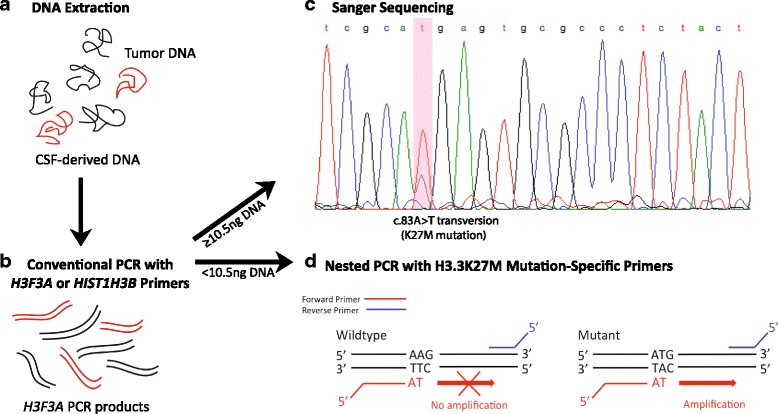
Experimental Design for H3 Mutation Detection. **a** DNA isolated from patient CSF may contain a small amount of tumor DNA (*red*). **b** PCR amplification of *H3F3A* or *HIST1H3B* was performed on all extracted DNA. **c** Specimens with ≥10.5 ng DNA were sequenced for c.83A > T mutation. **d** Specimens with <10.5 ng isolated DNA were submitted for a second round of PCR with primers designed to selectively amplify the *H3F3A* c.83A > T mutant allele, yielding a 150 bp product. *H3F3A* c.83A > T mutation results in lysine 27 codon transversion to methionine (AAG to ATG). The mutation-specific forward primer (*red*) is designed with the variant base (thymine) at the 3′ end, facilitating anchoring specificity to the mutant allele: this single nucleotide mismatch prevents wild type *H3F3A* amplification. Reverse primer complementary to the wild type sequence is indicated in *blue*. Schematic adapted from Zhang et al.[39]

Template DNA isolated from CSF, tumor tissue and tumor cells was amplified via PCR using *H3F3A* primers (0.8 μM) flanking a 300 base pair exonal region encoding Lys27 and Gly34 in Histone H3.3 (Fig. 1, Additional file 1: Table S1). In cases where adequate CSF volume was available (*n* = 2) and/or H3 status could not be confirmed by tissue analysis (*n* = 1), *H3F3A* wild type DNA specimens were subsequently subjected to PCR amplification with *HIST1H3B* primers (0.8 μM) flanking a 700 base pair exonal region encoding Lys27 in Histone H3.1 (Additional file 1: Table S1). Conventional PCR was performed in a thermocycler (Bio-Rad) under the following conditions: two minutes at 95 °C, 40 cycles of (25 s at 95 °C, 35 s at 55 °C, 40s at 72 °C), and five minutes at 72 °C. PCR products separated in 2% agarose gel and full-length *H3F3A* DNA purified using the QIAquick Gel Extraction Kit (Qiagen). Briefly, three volumes of buffer QG was added to one volume of gel (1 mg gel = 1 μL), and the mixture incubated at 50 °C for 10–15 min to melt the agarose. Isopropanol was added to the mixture (1 gel volume), and the gel-DNA mixture passed by the supplied spin column followed by one round of washing with buffer PE, and one round of dry spin to remove residual wash buffer. Finally, 25–30 μL buffer EB was used to elute the DNA. DNA was quantified using NanoDrop 2000 (Thermofisher), and submitted to Sanger sequencing of *H3F3A* or *HIST1H3B* for K27M mutation using the ABI 3730 High-Throughput DNA Sequencer (Applied Biosystems). For some tumor tissue, results of clinical next-generation sequencing were available. [24]. Sequenced data were visualized with FinchTV (Geospiza) and MegAlign (DNASTAR).

### Targeted H3.3K27M detection via nested PCR

Detection of the *H3F3A* c.83A > T transversion (H3.3K27M) in CSF specimens with less than 10.5 ng DNA was achieved using a strategy adapted from Zhang et al. [39] (Fig. 1d). An H3.3K27M mutation-specific forward primer with the 3′-end resting on the variant base of the mutant allele was used. Reverse primers complementary to the wild type sequence were designed and optimized using the NCBI Primer-BLAST tool to eliminate primer dimers and achieve an optimal length for 60 °C melting temperature (T_M_). All primer sets were designed to amplify 150 bp of the *H3F3A* gene. 10 ng of template DNA and 0.8 μM of primers were used in each PCR reaction. Thermocycling was performed in a PCR machine (Bio-Rad) under the following conditions: two minutes at 95 °C, 40 cycles of (25 s at 95 °C, 35 s at 55 °C, 40s at 72 °C), and five minutes at 72 °C. Two forward and eight reverse primers were designed, each varied in length by 18–21 basepairs. Primer screening was performed using DNA isolated from cell lines with known *H3F3A* mutation status. Primer pairs with the most robust mutation-specific results (*n* = 3) were selected for further analyses (Additional file 1: Table S1).

### Mutation validation via tissue immunohistochemistry

Tissue immunohistochemistry was performed as follows: serial sections of four microns were cut from paraffin blocks. Antigen retrieval was performed using 1× DAKO Target Retrieval Solution pH 6 (Dako S1699). Slides were incubated in Biocare Medical Decloaking Chamber at 110 °C for five minutes, followed by incubation in PBS for five minutes. Primary antibodies were prepared to a final volume of 200 μL using antibody diluent (Dako S0809) as follows: rabbit polyclonal anti-Histone H3K27M (Millipore ABE419) 1:1000 and rabbit monoclonal anti-Histone H3K27me3 (Cell Signaling Technology #9733) 1:100. Slides were incubated with primary antibody at 4 °C overnight then washed for three minutes in TBST (Dako S3306). Immunohistochemical reactions were visualized using DAB chromogen (Dako K4011). The slides were counter stained with hematoxylin for one minute at room temperature, washed with tap water and dehydrated with graded alcohol and xylene, and finally coverslip using a xylene-based mounting medium. Hematoxylin and Eosin (H&E) staining was performed on each specimen to validate tumor diagnosis by the Pathology Department at the Ann & Robert H. Lurie Children’s Hospital of Chicago.

## Results

### Comparison of nucleic acid precipitation carriers

**Fig. 2:**
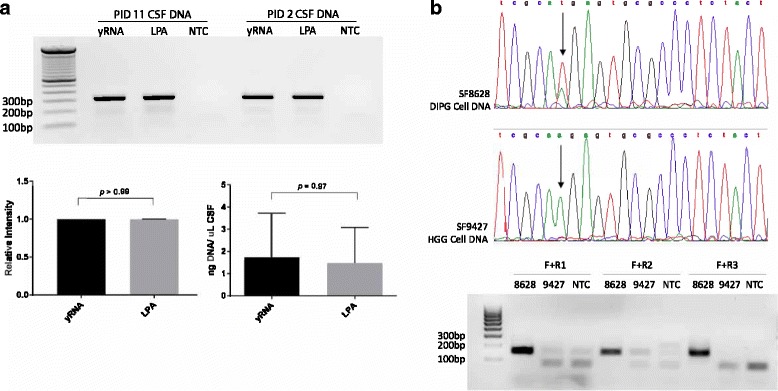
Selection of Precipitation Carriers and Mutation-Specific Primers. **a** The quantity and quality of DNA extracted from CSF using carrier RNA (yRNA) or linear polyacrylamide (LPA) were compared using matched CSF specimens (*n* = 4). PCR-amplification of *H3F3A* in CSF-derived DNA using yRNA and LPA yielded 300 bp bands at equivalent intensity (yRNA mean intensity normalized to 1; LPA mean relative intensity = 0.99; Mann-Whitney *U* test, *p* > 0.99, band intensities analyzed with ImageJ) with gel results from two specimens shown (PID 2 and 11). No significant difference was detected in the amount of DNA recovered per microliter CSF between the two carriers (yRNA mean = 1.74 ng DNA/μL CSF; LPA mean = 1.47 ng/μL CSF; Mann-Whitney *U* test, *p* = 0.97). **b** Prior to primer testing, *H3F3A* c.83 A > T mutation status of a DIPG cell line SF8628 (mutant) and pediatric glioblastoma (high-grade glioma, HGG) cell line SF9427 (wild type) was confirmed by Sanger Sequencing. Selective amplification of the mutant *H3F3A* allele in SF8628 was achieved using all three H3.3K27M primer pairs (Table 1)

To compare the efficacy of carrier RNA (yRNA) and linear polyacrylamide (LPA) as nucleic acid precipitation carriers, we used each carrier to extract DNA from matched CSF aliquots from four patients (Table 1). We observed no significant difference (*p* = 0.97) in Sanger sequencing results or the amount of nucleic acid extracted using carrier RNA (mean = 1.74 ng/μL CSF) compared to LPA (mean = 1.47 ng/μL CSF, Fig. 2a). These data demonstrate that LPA does not compromise the yield or quality of the nucleic acid isolated from CSF. Given our intent to investigate CSF-derived RNA in subsequent analysis, we used LPA for DNA extraction from all remaining CSF specimens (*n* = 8).

### Comparison of centrifugation conditions and extracted DNA fragment size

To evaluate the effect of CSF centrifugation on isolated DNA fragment size, CSF from PID 4 was centrifuged at three different conditions described in Methods, and the fragment distribution of isolated DNA was examined. As expected, smaller DNA fragments were isolated with greater centrifugation speed and duration. At 1000 *g* × 10 min, extracted DNA fragments were exclusively 150 bp, consistent with known size of cell-free circulating tumor DNA (ctDNA) released by apoptotic cells [29]. At lower spin speed and time, larger fragments were also observed (Additional file 2: Figure S3).

### Detection of H3 mutation in CSF-derived DNA

All CSF-extracted DNA underwent traditional PCR amplification of *H3F3A* (Fig. 1a, b). For CSF specimens containing <10.5 ng DNA (4/12, 33%, PIDs 1, 5, 6, 12.), the amount of PCR-amplified *H3F3A* DNA was not sufficient in quality and quantity to subsequently undergo Sanger sequencing. To circumvent this problem, we employed a nested PCR strategy based on previously described methods [39]. After two rounds of 40-cycle PCR amplification with *H3F3A* primers as described above, the resultant pool of *H3F3A* genes (300 bp) were subjected to a second round of PCR with *H3F3A* c.83A > T (H3.3K27M) mutation-specific primers (Fig. 1d). One forward and eight reverse primers were designed. Primer specificity was tested using genomic DNA isolated from pediatric glioma cell lines SF8628, a DIPG cell line harboring the H3.3K27M mutation, and SF9427, a H3 wildtype supratentorial high-grade glioma cell line (Fig. 2b). Of the eight primer pairs, three were determined to be most selective for the mutation (F + R1, R2, R3) (Fig. 2b, Additional file 1: Table S1). Reverse primer 3 (R3) yielded the cleanest selective amplification between the mutant and wildtype cell lines, and thus was utilized for all subsequent analyses. CSF from a patient with congenital hydrocephalus with no history of brain tumor (PID 12) was included as a negative control for mutation-specific primer testing (Additional file 3: Figure S1).

**Fig. 3:**
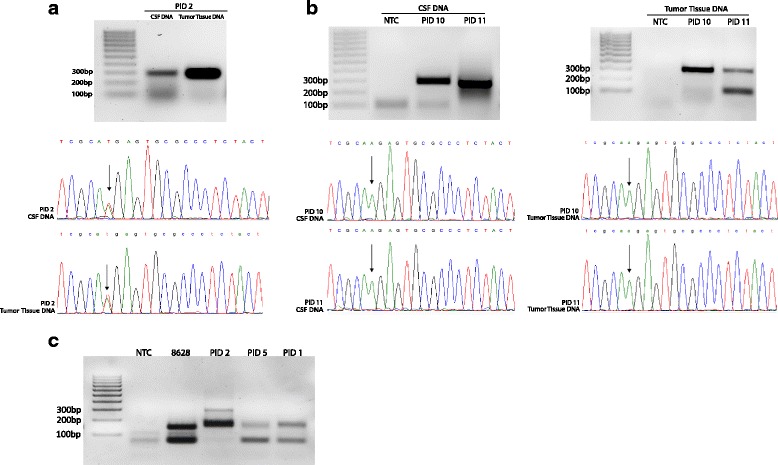
H3K27M Detection and Validation in Patient CSF and Tumor Tissue Specimens. **a** CSF-derived DNA and DNA from matched fresh frozen DIPG tumor tissue (PID 2) was submitted for PCR-amplification of a 300 bp region of *H3F3A* for mutation detection*.* Sanger sequencing chromatograph of resulting PCR-amplified *H3F3A* confirmed c.83A > T transversion in CSF DNA and matched DIPG tumor tissue DNA (*arrow*). **b** CSF-derived DNA and matched fresh frozen paraffin embedded (FFPE) tumor tissue from PID 10 and 11 was submitted for PCR-amplification of a 300 bp region of *H3F3A* for mutation detection*.* Sanger sequencing of resulting PCR-amplified *H3F3A* from CSF and FFPE tumor tissue demonstrated absence of mutation. **c** Targeted *H3F3A* c.83A > T amplification using CSF-derived DNA from PID 1 and 5 demonstrated presence of mutation, with DNA from H3.3K27M DIPG tissue (PID 2) and primary tumor cells (SF8628) as positive controls

For CSF specimens containing ≥10.5 ng DNA (8/12, 66.7%, PIDs 2–4, 7–11), traditional Sanger sequencing after PCR amplification of *H3F3A* was employed to detect the c.83A > T transversion (Figs. 1c and 3a, b). Two *H3F3A* wild type specimens with sufficient extracted DNA were subsequently submitted for *HIST1H3B* PCR amplification and Sanger sequencing to detect the H3.1K27M mutation (PIDs 3, 10). Of the eight CSF specimens analyzed with this technique, *H3F3A* c.83A > T (H3.3K27M) was detected in two of four DIPG CSF specimens (PID 2, 4). This result was confirmed in matched fresh frozen tumor tissue via Sanger sequencing (Fig. 3a). H3.3K27M was not detected in the one DIPG CSF specimens tested with this technique (PID 3). H3.1K27M mutation was also not detected in CSF-derived DNA from PID 3 via this technique, and matched tumor tissue was not available for sequencing or immunohistochemical analysis. As expected, neither H3.3K27M nor H3.1K27M was detected in CSF from patients harboring non-midline CNS tumors (PID 7–11, Fig. 3b, Table 1). H3.3G34V was detected in CSF-derived DNA from one patient in our cohort with a hemispheric glioblastoma with thalamic extension (PID 8, Table 1).

Targeted *H3F3A* c.83A > T mutation amplification was performed on CSF specimens containing <10.5 ng DNA, including three brain tumor patients (PID 1, 5 and 6), and one child with congenital shunted hydrocephalus but no history of brain tumor (PID 12). H3.3K27M was identified in PID 5 (thalamic anaplastic astrocytoma) and PID 1 (DIPG), while the low quantity of extracted DNA from the remaining DIPG specimen (PID 6) precluded further analysis (Table 1, Fig. 3c). In cases with ample amount of DNA remaining after Sanger sequencing (PIDs 2–4), targeted mutation amplification was also performed to test concordance between the two methods. We found our two methods to be 100% concordant (Additional file 4: Figure S4). Additionally, to ensure that primer specificity was not affected by the source or amount of input DNA, we confirmed H3.3K27M detection using F + R3 primers in DNA from DIPG patient PID 2, as well as in the *H3F3A* gene pool amplified from genomic DNA of H3.3K27M mutant DIPG cell line SF8628 (Fig. 3c). To ensure our mutation-specific primers did not exhibit non-specific DNA binding, CSF-derived DNA from a child with congenital shunted hydrocephalus (PID 12) was also analyzed, with no amplification product identified, as expected (Additional file 3: Figure S1). Overall, of the six patients in our cohort with diffuse midline glioma (expected to harbor an H3K27M mutation), sufficient DNA for sequencing was isolated from five (83.3%), with H3.3K27M mutation detected in four (67%), including 3/4 DIPGs and 1/2 thalamic anaplastic astrocytomas.

### Validation of H3K27M in tumor tissue

**Fig. 4:**
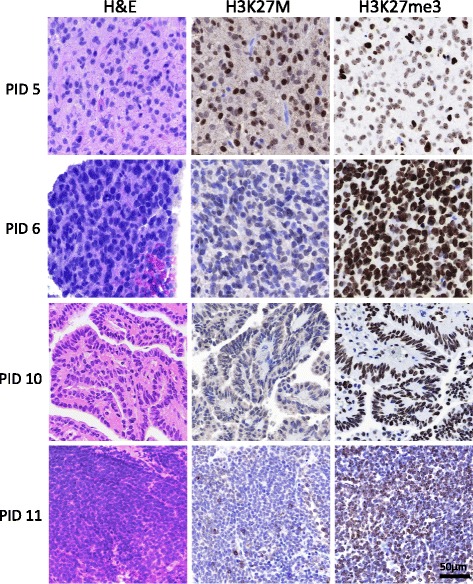
H3K27M and H3K27me3 Tissue Immunohistochemical Staining. Immunohistochemical staining for H3K27M and H3K27me3 was evaluated in tumor tissue specimens (*n* = 7). H3K27M staining patterns were consistent with CSF and tumor tissue sequencing results. Decreased H3K27me3 was observed in H3.3K27M mutant tumor tissue (PID 5), relative to wild type specimens (PID 6, 10, 11). Similar results were observed in PID 8 and 9 (data not shown). Tumor histologic diagnosis was confirmed with Hematoxylin and Eosin staining. Scale bar = 50 microns

To validate results of our CSF-derived DNA analysis, H3K27M and H3K27me3 (H3K27 trimethylation) were evaluated via immunohistochemical staining of available matched tumor tissue specimens (*n* = 7, Table 1, Fig. 4). As expected, H3K27M staining (which detects both H3.1 and H3.3K27M) was positive in tumor tissue PID 5 (thalamic anaplastic astrocytoma), consistent with CSF DNA sequencing results (Fig. 3c). Decreased H3K27me3 was also observed in PID 5 tumor tissue, consistent with previous reports of global decrease in H3K27 trimethylation in H3K27M tumors [18, 35]. Positive H3K27M and decreased H2K27me3 was also observed in PID 4 tumor tissue (data not shown), in concordance with tissue DNA sequencing results. Conversely, non-midline tumor tissue specimens PID 6, 10, 11 (Fig. 4) 8 and 9 (data not shown) demonstrate negative H3K27M staining, consistent with corresponding CSF sequencing results, and heavy H3K27me3 positivity, as expected. H3K27 wild type status was confirmed in these specimens via Sanger sequencing of DNA extracted from FFPE tumor tissue (Fig. 3b).

## Discussion

We present the first report of Histone H3 mutation detection in CSF from children with diffuse midline glioma. Given the high frequency and significant biological implications of histone H3 mutation in these tumors, “liquid biopsy” via CSF analysis may serve as an important approach for H3 mutation detection to impact patient treatment. Indeed, pre-clinical evaluation of agents aimed at the downstream effects of H3K27M in diffuse midline glioma demonstrate efficacy [11, 12, 27], and biopsy-based clinical trials for patient stratification to molecularly targeted treatments based on H3 mutation status are now underway [15, 17, 36]. However, while recent advances in neurosurgical and imaging techniques have made tumor biopsy for genetic analysis technically feasible, tissue acquisition from the brainstem or thalamus is not without risk, and brainstem glioma biopsy is not yet routinely performed. In contrast, CSF is more safely accessible than midline brain tumor tissue, and may provide a more accurate representation of mutation status than small tissue specimens. For example, tumor-specific mutations can be detected and quantified in CSF-derived tumor DNA from patients with primary and metastatic brain tumors as a correlate of tumor burden for clinical diagnosis and measuring tumor response to therapy [19, 25, 26, 34, 37]. Our results demonstrate the feasibility and specificity of H3K27M detection in DNA from CSF from children with diffuse midline glioma, and suggest the potential clinical utility of CSF analysis for determining H3 mutation status in these patients.

We selected available archival CSF specimens collected from a cohort of pediatric brain tumor patients (*n* = 11) for Histone H3 mutation detection. An additional CSF specimen from a child with congenital hydrocephalus and no history of brain tumor was analyzed as a negative control. Specimen selection was based on tumor histopathologic diagnosis, location, grade, site and time of CSF acquisition, and specimen availability. Given previous reports we anticipated a low yield of DNA from CSF specimens. Therefore, in order to maximize the likelihood of H3 mutation detection, CSF specimens from children with intraventricular tumors (*n* = 2) and CSF diversion devices in close proximity to tumor tissue (*n* = 5) were preferentially selected for study. Diffuse midline glial tumors (PID 1–6) were hypothesized to have a higher likelihood of H3K27M mutation, while the remaining specimens in our cohort studied were expected to be H3.3G34V (PID 8) or H3 wild type (PID 7, 9–12). H3K27M status was evaluated matched tumor tissue when available (*n* = 8) to validate the sensitivity and specificity of CSF analysis for mutation detection.

In order to develop a robust, reliable method for H3 mutation detection in CSF, we first sought to identify the most suitable precipitation carrier for nucleic acid extraction. In an RNA analysis workflow, both the extracted target mRNA and carrier RNA is subjected to reverse transcription and second-strand synthesis, which can confound downstream analysis and library construction for RNA-sequencing. To our best knowledge, there is no effective way to isolate carrier RNAs from target mRNAs. While the Illumina Truseq RNA preparation workflow can be used to purify poly-A containing mRNA molecules using poly-T oligo-attached magnetic beads, this approach is not effective for isolating carrier RNAs, as these also contain poly-A tails. Size selection also cannot be used to isolate carrier RNA (yRNA), as the carrier is often several orders of magnitude longer than extracted nucleic acids of interest. We therefore compared linear polyacrylamide (LPA) as an alternative to carrier RNA [1, 9, 13], and demonstrate that LPA is as effective as carrier RNA for nucleic acid precipitation. Given our intent to investigate CSF-derived RNA, we used LPA for all subsequent CSF DNA extractions.

In order to determine the source of DNA isolated from CSF specimens in our cohort (genomic tumor DNA or cell-free ctDNA), we evaluated extracted DNA fragment size. Our data demonstrate that centrifugation at 1000 *g* × 10 min is sufficient to isolate 150 bp DNA fragments, consistent with cell-free circulating tumor DNA (ctDNA). Our results also suggest CSF specimens in the present cohort contain a mixture of both genomic tumor DNA and ctDNA (Additional file 2: Figure S3). However, if quantifying changes in H3 mutation frequency, for diagnosis or monitoring disease progression or response to treatment, it is necessary to distinguish the source of DNA isolated in CSF specimens [29]. Further studies to preferentially isolate ctDNA from CSF specimens submitted for H3 mutation analysis are therefore warranted, and currently underway.

Overall, DNA was isolated from all CSF specimens studied (*n* = 12). In 8/12 specimens, DNA yield was sufficient for sequencing of amplified *H3F3A* gene product for c.83A > T and c.104G > T transversion. In two *H3F3A* wild type cases with sufficient DNA for further testing, *HIST1H3B* sequencing for c.83A > T transversion was also performed. Sanger sequencing can detect histone H3 point mutations with precision, without the need for negative controls [39], but does require a threshold quantity and quality of gene fragments to ensure the predominant wild type allele does not mask the mutant signal to yield a false-negative result. In our study, the DNA yield from 4/12 CSF specimens was below this threshold (<10.5 ng). Rather than applying multiple rounds of PCR amplification to these specimens, a nested-PCR strategy was employed for selective amplification of H3.3K27M mutant *H3F3A* alleles from a total pool of *H3F3A* in order to prevent amplification bias of smaller-sized DNA fragments [6]. For this approach, a forward H3.3K27M mutation-specific primer was designed with the 3′-end anchoring to the variant-base of the mutant allele (Fig. 1d), to ensure that only the allele containing the missense mutation would be elongated and amplified, minimizing the likelihood of a false negative result. Three reverse primers, all complementary to the wild type sequence, were also designed to amplify 150 bp of the *H3F3A* gene. Using this approach, we detected H3.3K27M in two additional CSF specimens from patients with midline glioma. This strategy is therefore an effective alternative sequencing approach when working with a very low amount of starting nucleic acid and for detecting mutations with low starting allelic frequency, as the primer is highly mutation-specific. In three DIPG cases with sufficient amount of DNA isolated, both H3K27M detection strategies were performed and the results were found to be 100% concordant.

The described method for H3K27M detection in CSF-derived DNA effectively circumvents a major challenge in detecting an oncogenic mutation: low relative concentration of mutant DNA in source material. Because oncogenic mutations occur in only 0.1–1% of all DNA molecules for a given genomic locus, deep sequencing coverage is required to achieve sufficient sensitivity for detection [26]. Our cohort included CSF specimens from seven children harboring diffuse midline gliomas (Table 1). We detected the H3 mutation in 66.7% (4/6) of CSF-derived DNA analyzed, including three DIPGs (H3.3K27M) and one thalamic anaplastic astrocytoma. Analysis of one additional CSF specimen from diffuse midline glioma (PID 3) did not reveal H3.3 or H3.1 K27M mutation, while sequencing of CSF from PID 6 was not possible due to the low quantity of starting DNA (<0.5 ng). As expected, all CSF specimens from children with tumors located outside midline were negative for H3K27M, and H3.3G34V was detected in CSF from the patient with supratentorial glioblastoma (PID 8). H3K27M status was validated in tumor tissue (*n* = 8) via IHC staining (*n* = 7) and/or genetic sequencing (*n* = 4). Tissue analysis results were 100% concordant with DNA analysis results of H3.3 K27M status (Table 1) for cases in which both analyses were possible (*n* = 6). The lack of available tumor tissue for analysis from three patients in our cohort (PID 1, 3 and 7) underscores the need for an alternative technique for H3 mutation detection in children harboring midline glioma. Finally, since global loss of H3K27 trimethylation is associated with H3K27M mutation, tumor tissue specimens were also stained H3K27me3. Results were consistent with expected relative patterns of K27 post-translational modification in H3 mutant and wild type tumors, providing additional validation of our CSF mutation analysis results.

We postulate that timing, technique and location of CSF collection may influence test sensitivity, and must therefore be considered when interpreting CSF DNA results. Importantly, a higher concentration of CSF-derived DNA was isolated from patients with intraventricular tumor extension and/or from CSF collected from ventricles in close anatomic proximity to tumor tissue (PID 5–8, 10–11; mean = 1.0 ng/μL CSF), compared to CSF collected from the lateral ventricle in patients with posterior fossa or brainstem tumors (PID 1–4, 9; mean = 0.16 ng/μL CSF, Additional file 5: Figure S2). Of note, the CSF specimens in our cohort were archival and hence not preserved in nuclease-free tubes. Given that low starting DNA can limit mutation detection, we have subsequently collected tumor tissue and CSF specimens from adult and pediatric brain tumor patients using nuclease-free tubes for increased mutation detection sensitivity using a recently published next generation sequencing technique [24].

Importantly, the H3K27M mutation allelic frequency in midline glioma tissue is thought to be between 15 and 58% [8, 23]. Likewise, CSF-derived DNA has a lower proportion of wild type DNA relative to the mutant form. However, the described technique of DNA amplification and sequencing achieves high specificity for H3K27M mutant allele detection, without the need for costly next-generation sequencing. Given the emerging evidence of spatial and temporal heterogeneity of diffuse midline glioma tissue, coupled with the small volume of tissue typically acquired via stereotactic needle tumor biopsy, selective amplification of tumor DNA from CSF specimens may serve as a superior method for clinical detection of H3 mutation in this patient population. Indeed, detection of somatic mutations in CSF collected by lumbar puncture from brain tumor patients via droplet digital PCR (ddPCR) and targeted amplicon sequencing, with utility for monitoring tumor burden and response to therapy, was recently reported [25, 26]. Comparison of perilesional cisternal, ventricular and lumbar CSF collected from patients with diffuse midline glioma, using mutation-specific primers and a ddPCR technique, could therefore be of value for determining the threshold DNA concentration and optimal source of tumor DNA for H3 mutation detection. In turn, CSF acquisition for longitudinal quantitative H3 mutation detection, via serial reservoir taps or lumbar puncture in lieu of tumor tissue sampling, could be of utility for measuring tumor burden and response to molecularly-directed treatments in these patients.

Overall, our results demonstrate the feasibility of histone H3 mutation detection in DNA derived from archival CSF from children with brain tumors. In our patient cohort, the described technique was 100% specific for H3K27M detection in patients with diffuse midline glioma, with 87.5% sensitivity (in specimens for which matched tissue was available for validation). These data suggest that CSF collection for H3 mutation analysis may be considered for patients with diffuse midline glioma, including DIPG, for diagnosis and to potentially facilitate stratification to molecularly-directed cancer therapy without the need for tumor tissue.

## Conclusion

We report the successful detection of histone H3 mutations from children with brain tumors, including high-grade and diffuse midline glioma, using archival CSF specimens. We present two analytic techniques for H3 mutation analysis: Sanger sequencing of CSF-derived tumor DNA, and nested PCR of DNA using mutation-specific primers. Both strategies are cost-efficient, mutation specific, and clinically feasible, and CSF collection has significantly less clinical morbidity than brainstem or thalamic tumor tissue biopsy. Given the midline anatomic location and genetic heterogeneity of these tumors, CSF may prove a superior substrate for clinical H3K27M detection compared to small stereotactic biopsy specimens. Our data suggest that H3 mutation analysis of CSF-derived DNA from children with diffuse midline glioma may be useful for “liquid biopsy” in order to facilitate diagnosis, enable patient stratification to molecularly-targeted therapy, monitor response to treatment, and ultimately improve patient outcomes for this devastating disease.

## Additional files

Additional file 1: Table S1. List of Histone H3 Primer Sequences. The sequences and properties of primers used in this study are described. Melting temperature (T_M_) was calculated at 0.4 μM.fdg. (XLS 22 kb)

Additional file 2: Figure S3. Effect of Centrifugation on CSF-Extracted Nucleic Acid Fragment Size. CSF from one DIPG patient (PID4) was centrifuged under three different conditions and resulting isolated DNA fragment size distribution was measured via electrophoresis. Conditions are as indicated in the table and results depicted in corresponding electrophorogram for each condition set. Smaller DNA fragments of 160 bp, consistent with ctDNA, were isolated with greater centrifugation speed and duration. (PDF 1487 kb)

Additional file 3: Figure S1. Negative control for mutation-specific primers. CSF from a patient with congenital hydrocephalus and no history of brain tumor (PID 12) was analyzed using *H3F3A* c.83A > T mutation-specific primers to demonstrate primer specificity, with DNA from primary tumor cells (SF8628) as a positive control. (PDF 414 kb)

Additional file 4: Figure S4. H3K27M Detection in Patient CSF via Sanger Sequencing and Targeted Mutation Amplification. CSF-derived DNA from DIPG patients PID 3 and 4 was submitted for PCR-amplification of a 300 bp region of *H3F3A* for mutation detection. Sanger sequencing chromatograph of resulting PCR-amplified *H3F3A* depicts c.83A > T transversion in CSF-derived DNA from PID 4, but not PID 3. These results were confirmed with targeted *H3F3A* c.83A > T amplification via nested PCR. CSF-derived DNA from DIPG patient PID 2 previously confirmed to harbor H3.3K27M is included as positive control; CSF-derived DNA from PID 12 with congenital hydrocephalus previously confirmed to be H3.3 wild type is included as negative control. (PDF 1429 kb)

Additional file 5: Figure S2. CSF DNA Yield Relative to Tumor Location. Greater DNA concentration (ng/μL) was extracted from lateral ventricular CSF from patients with interventricular tumors or tumors adjacent to the lateral ventricle, in comparison to patients with tumors in a distant or non-adjacent anatomic location (Adjacent mean = 1.00 ng DNA/μL CSF; Distant mean = 0.16 ng/μL CSF; Mann–Whitney *U* test, *p* = 0.39). Scatter plot whiskers: mean with standard deviation. (PDF 481 kb)

## Abbreviations

CSF: Cerebrospinal fluid
ctDNA: Cell-free circulating tumor DNA
ddPCR: Droplet digital polymerase chain reaction
DIPG: Diffuse intrinsic pontine glioma
EVD: External ventricular drain
FFPE: Fresh-frozen paraffin embedded
gDNA: genomic DNA
H&E: Hematoxylin and Eosin
LPA: Linear polyacrylamide
MRI: Magnetic resonance imaging
yRNA: yeast RNA
