# Supplementary material for: Detection of Histone H3 mutations in cerebrospinal fluid-derived tumor DNA from children with diffuse midline glioma
Source: Acta Neuropathol Commun. 2017 Apr 17;5:28. doi: 10.1186/s40478-017-0436-6 (PMC5392913; doi:10.1186/s40478-017-0436-6)

Figure S3.

Centrifugation Conditions	Speed (x *g*)	Duration (minutes)	DNA Fragment Length (bp)
1	0	0	> 2000
2	500	5	> 151
3	1000	10	150

Condition 1

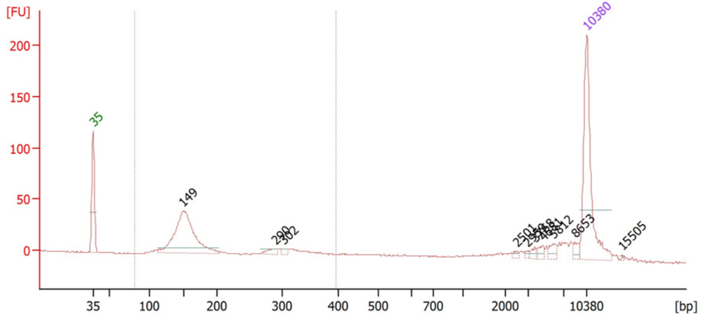

Condition 2

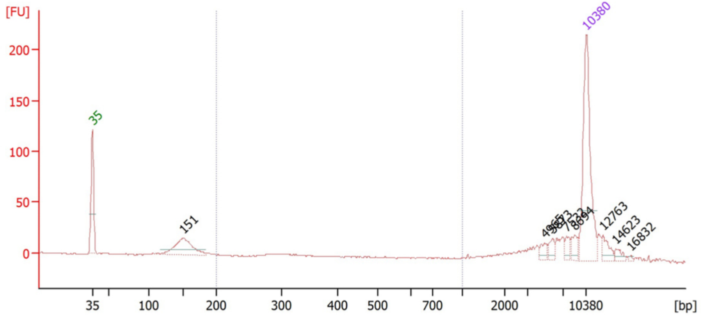

Condition 3

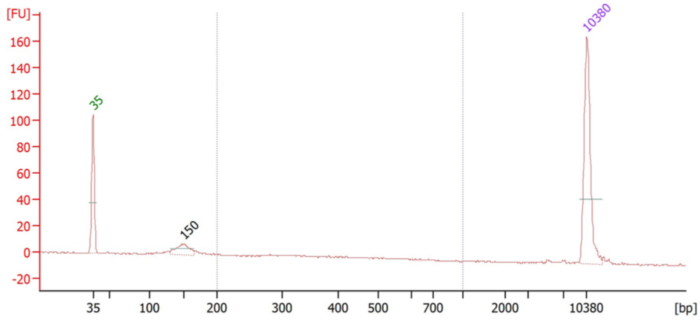

Supplement: Supplementary file 2 — Effect of Centrifugation on CSF-Extracted Nucleic Acid Fragment Size. CSF from one DIPG patient (PID4) was centrifuged under three different conditions and resulting isolated DNA fragment size distribution was measured via electrophoresis. Conditions are as indicated in the table and results depicted in corresponding electrophorogram for each condition set. Smaller DNA fragments of 160 bp, consistent with ctDNA, were isolated with greater centrifugation speed and duration. (PDF 1487 kb) [file 40478_2017_436_MOESM2_ESM.pdf]
