# Supplementary figures and images for: Detection of Histone H3 mutations in cerebrospinal fluid-derived tumor DNA from children with diffuse midline glioma
Source: Acta Neuropathol Commun. 2017 Apr 17;5:28. doi: 10.1186/s40478-017-0436-6 (PMC5392913; doi:10.1186/s40478-017-0436-6)

Figure S1.

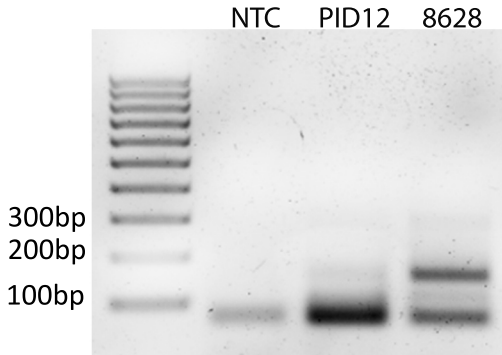

KEY: NTC = no template control

Supplement: Supplementary file 3 — Negative control for mutation-specific primers. CSF from a patient with congenital hydrocephalus and no history of brain tumor (PID 12) was analyzed using H3F3A c.83A > T mutation-specific primers to demonstrate primer specificity, with DNA from primary tumor cells (SF8628) as a positive control. (PDF 414 kb) [file 40478_2017_436_MOESM3_ESM.pdf]

Figure S4.

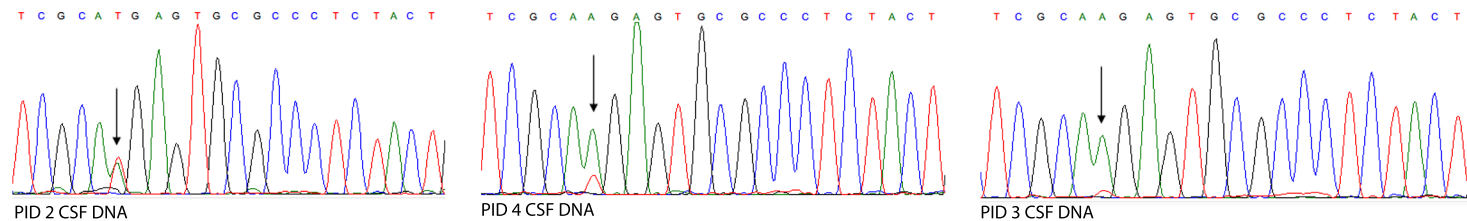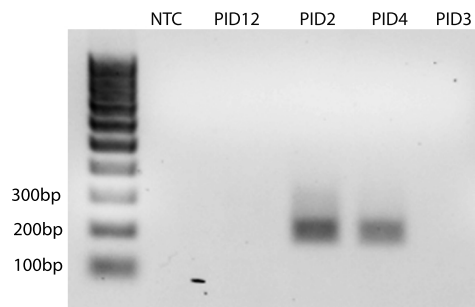

KEY: NTC = no template control; CSF DNA = CSF-derived DNA

Supplement: Supplementary file 4 — H3K27M Detection in Patient CSF via Sanger Sequencing and Targeted Mutation Amplification. CSF-derived DNA from DIPG patients PID 3 and 4 was submitted for PCR-amplification of a 300 bp region of H3F3A for mutation detection. Sanger sequencing chromatograph of resulting PCR-amplified H3F3A depicts c.83A > T transversion in CSF-derived DNA from PID 4, but not PID 3. These results were confirmed with targeted H3F3A c.83A > T amplification via nested PCR. CSF-derived DNA from DIPG patient PID 2 previously confirmed to harbor H3.3K27M is included as positive control; CSF-derived DNA from PID 12 with congenital hydrocephalus previously confirmed to be H3.3 wild type is included as negative control. (PDF 1429 kb) [file 40478_2017_436_MOESM4_ESM.pdf]

Figure S2.

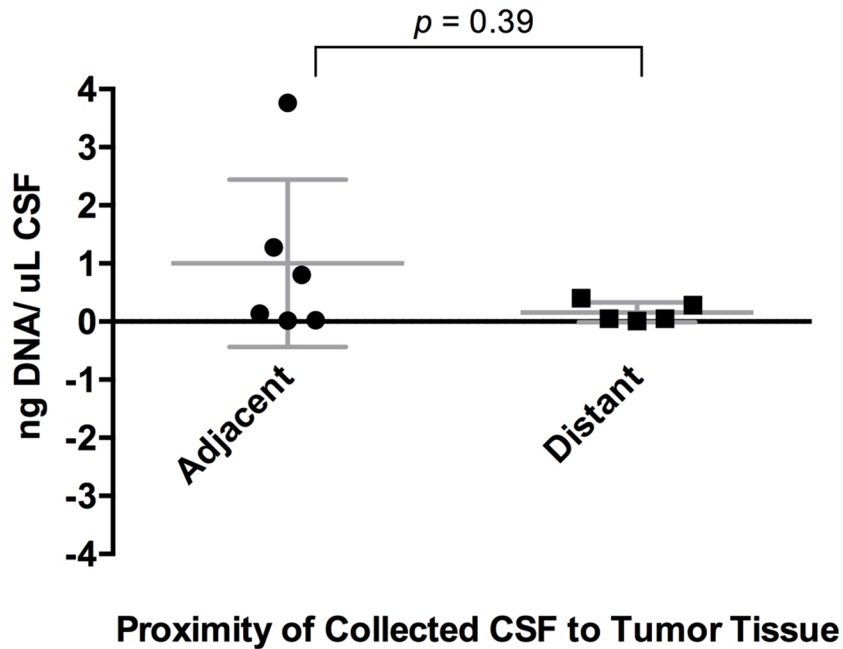

Supplement: Supplementary file 5 — CSF DNA Yield Relative to Tumor Location. Greater DNA concentration (ng/μL) was extracted from lateral ventricular CSF from patients with interventricular tumors or tumors adjacent to the lateral ventricle, in comparison to patients with tumors in a distant or non-adjacent anatomic location (Adjacent mean = 1.00 ng DNA/μL CSF; Distant mean = 0.16 ng/μL CSF; Mann–Whitney U test, p = 0.39). Scatter plot whiskers: mean with standard deviation. (PDF 481 kb) [file 40478_2017_436_MOESM5_ESM.pdf]
